# The Mediating Role of Patients’ Trust Between Web-Based Health Information Seeking and Patients’ Uncertainty in China: Cross-sectional Web-Based Survey

**DOI:** 10.2196/25275

**Published:** 2022-03-11

**Authors:** Wei Dong, Xiangxi Lei, Yongmei Liu

**Affiliations:** 1 School of Business Central South University Changsha China; 2 Department of Information Systems City University of Hong Kong Hong Kong Hong Kong; 3 China Mobile Group Hunan Company Limited Changsha China

**Keywords:** patient trust, online health information quality, online word-of-mouth, patient uncertainty, principal-agent theory, physician-patient relationship

## Abstract

**Background:**

In the physician-patient relationship, patients’ uncertainty about diseases and the lack of trust in physicians not only hinder patients’ rehabilitation but also disrupt the harmony in this relationship. With the development of the web-based health industry, patients can easily access web-based information about health care and physicians, thus reducing patients’ uncertainty to some extent. However, it is not clear how patients’ web-based health information–seeking behaviors reduce their uncertainty.

**Objective:**

On the basis of the principal-agent theory and the perspective of uncertainty reduction, this study aims to investigate the mechanism of how web-based disease-related information and web-based physician-related information reduce patients’ uncertainty.

**Methods:**

A web-based survey involving 337 participants was conducted. In this study, we constructed a structural equation model and used SmartPLS (version 3.3.3; SmartPLS GmbH) software to test the reliability and validity of the measurement model. The path coefficients of the structural model were also calculated to test our hypotheses.

**Results:**

By classifying patients’ uncertainties into those concerning diseases and those concerning physicians, this study identified the different roles of the two types of patients’ uncertainty and revealed that web-based disease-related information quality and web-based physician-related information can act as uncertainty mitigators. The quality of disease-related information reduces patients’ perceived information scarcity about the disease (β=−.588; *P*<.001), and the higher the information scarcity perceived by patients, the higher their uncertainty toward the disease (β=.111; *P*=.02). As for physician-related information, web-based word-of-mouth information about physicians reduces patients’ perceived information scarcity about the physician (β=−.511; *P*<.001), mitigates patients’ fears about physician opportunism (β=−.268; *P*<.001), and facilitates patients’ trust (β=.318; *P*<.001). These factors further influence patients’ uncertainty about the physician. In addition, from the test of mediating effect, patients’ trust in the physician fully mediates the relationship between their perceived information scarcity about the physician’s medical service and their uncertainty about the physician. Patients’ trust also partially mediates the relationship between their fear of the physician’s opportunism and their uncertainty about the physician. As for the two different types of uncertainty, patients’ uncertainty about the physician also increases their uncertainty about the diseases (β=.587; *P*<.001).

**Conclusions:**

This study affirms the role of disease-related web-based information quality and physician-related web-based word-of-mouth information in reducing patients’ uncertainties. With regard to the traits of principal-agent relationships, this study describes the influence mechanism based on patients’ perceived information scarcity, fears of physicians’ opportunism, and patients’ trust. Moreover, information about physicians is effective in reducing patients’ uncertainties, but only if the information enhances patients’ trust in their physicians. This research generates new insights into understanding the impact of web-based health information on patients’ uncertainties.

## Introduction

### Background

In a physician-patient relationship, it is always difficult for patients to evaluate medical services and their physicians because medical services are typical credence products [1,2]. Patients also lack the specialized knowledge to judge whether a physician’s treatments would be helpful before the treatments begin. Therefore, as principals, in this typical principal-agent relationship, patients face many uncertainties.

Previous research has explored how to achieve better treatment outcomes by reducing patients’ uncertainty [3-6]. The uncertainty in this principal-agent relationship is caused by information problems [7], such as hiding information and hiding behaviors; therefore, to reduce patients’ uncertainties, it is important to provide patients with more information. With the rapid development of patient-centered care [8], the physician-patient relationship is gradually changing from the traditional physician-led model to a new type of patient-centered diagnosis and treatment, with increasing emphasis on the role of patients [9]. The role of patients is changing from passive information recipients to active participants in medical decision-making [10]. The development of the eHealth industry has led to an increase in the number of patients who become electronic patients, namely, e-patients [9]. The channels for e-patients to obtain information about diseases and physicians have expanded, and this information can enhance patient-centered care [8]. For example, in a survey by Wong and Cheung [11], 97.32% (1162/1194) of the respondents used the internet, of which 87.44% (1016/1162) had used the internet to find health information. In a survey by Hedges and Couey [12], 90% of patients used web-based reviews to evaluate their physicians. By actively acquiring information about diseases and physicians through electronic information technology, e-patients can enhance their understanding of their medical condition and have a sense of control over their health, while reducing their uncertainties about the consultation processes and the physicians.

Although web-based information can reduce patients’ uncertainty to some extent, information overload can pose a major challenge [13], leading to confusion in e-patients. Incorrect information does not effectively reduce patients’ uncertainty. Moreover, this information may undermine patients’ trust and have a counterproductive effect [14]. Therefore, it is important to understand how patients’ web-based information consumption reduces their uncertainty, so that information providers can improve the design of information to better help patients. With this as the objective, this study intends to answer the following research questions: how do patients’ web-based information-seeking behaviors reduce their uncertainties about diseases and physicians? In addition, how does web-based information, such as information related to diseases and physicians, alleviate problems in this principal-agent relationship and then reduce patients’ uncertainty?

To address these research questions, based on the framework of *uncertainty mitigator–uncertainty antecedent–uncertainty*, this study explores how web-based health information mitigates patients’ uncertainty. The contributions of this study are as follows. First, based on the principal-agent theory and the uncertainty reduction theory (URT), this study explores the mechanism of how patients’ web-based health behaviors can reduce their uncertainty. Second, following the classification of consumers’ uncertainty about products and sellers by Dimoka et al [15], this study also distinguishes between patients’ uncertainty about diseases and physicians; the influence chain is also investigated. Finally, this study emphasizes the significant role of trust. Additional information can help reduce patients’ uncertainty, but only if it can enhance patients’ trust in their physicians.

### Principal-Agent Theory

Originating from the field of enterprise management, the principal-agent theory describes the relationship in which one entity (the principal) delegates work to another entity (the agent) who performs the work under a mutually agreed contract [16]. The relationship between enterprise owners and professional managers is a typical principal-agent relationship. This relationship applies to all transactional relationships in socioeconomic systems where opportunism, information asymmetry, and limited rationality exist. Owing to the separation of ownership and management rights of enterprises, the goals of principals and agents are inconsistent, which will lead to adverse selection before the contract [17] and the moral hazard of hidden behaviors after the contract [18].

The physician-patient relationship is also a typical principal-agent relationship in which the physician acts as an agent to provide medical services to the patient (the client) under a contract [19,20]. Patients, as principals, receive diagnoses of the disease, treatment plans, and medical care services from the agents (ie, physicians). Physicians and patients have inconsistent goals and asymmetrical information. Compared with physicians, patients are always at a disadvantage in information about diseases and physicians’ medical services. Patients want to receive superior medical services at a low cost to improve their health, whereas physicians want to provide medical services at a higher fee and lower cost (to themselves) to increase their income and reputation.

### Perceived Information Scarcity

Owing to the principal-agent relationship and the specialization of medical services, there is natural information asymmetry between physicians and patients [21]. Compared with physicians, patients have limited information about diseases and physicians, leading to patients’ perception of information scarcity. Previous literature defined scarcity as the limitation or unavailability of objects (eg, commodity) [22]. In the research of Wells et al [23], an individual’s degree of prepurchase information scarcity related to the product of interest is operationalized as whether a consumer had any prior information or experience with products offered on web-based shopping websites. Compared with physicians, patients lack professional medical education process and clinical experience; therefore, patients will be aware of the information scarcity regarding diseases and the physician’s medical service. In this study, patients’ perceived scarcity of information about diseases is defined as patients’ perception of their limited information related to diseases, whereas perceived scarcity of information about the physician’s medical service information is defined as patients’ perception of their limited information related to the physician’s medical service.

In the web-based environment, the emergence of information systems can help alleviate the principal–agent problem to some extent. For example, the website and product information can reduce customers’ information scarcity about products, thereby reducing customers’ worries about the platform’s opportunism and their purchase uncertainty [24]; the implementation of information systems within organizations, such as hospitals, was also found to be an effective means of improving information transparency [25]. Similarly, the disease-related and physician-related information obtained by patients through web-based searches can respectively help patients understand diseases and their physicians better. Web-based disease-related and physician-related information can reduce patients’ perceived scarcity of information about diseases and their physicians.

However, the information quality is unevenly distributed in the problem of information asymmetry [24], but existing studies failed to take into account the impact of the information quality of search behavior, especially because web-based health information lacks accuracy and credibility [26]. Information quality is always measured by the perceived information quality, which represents information receivers’ subjective perception about four dimensions of information quality, namely, relevance, adequacy, usefulness, and understandability of the information [27]. Higher-quality information can lead to better descriptions about the targets, and it is more useful than lower-quality information [28]. With a higher quality of diseases information in the web-based environment, patients will perceive the information as more relevant, adequate, and useful, thereby increasing their information about the diseases. As a result, the higher the quality of disease-related information sought by patients, the lower the perceived scarcity of information about the disease, leading to the following hypothesis: web-based health information quality reduces patients’ perceived scarcity of information regarding diseases (H1).

In addition to disease-related information, web-based health information provides patients with physician-related information, such as web-based word-of-mouth information about physicians, which represents other patients’ visiting experiences. In traditional offline hospitals, patients had very limited access to physicians’ medical service information, which was often confined to the small reach of word-of-mouth communication, making it difficult to obtain a large amount of word-of-mouth physician information. Web-based word-of-mouth information can effectively reduce asymmetries of products information [29,30]. Web-based word-of-mouth information can inform later customers about the details of the products or the service [28,31]. Similarly, physicians’ web-based ratings are also found to reflect their quality perceived by offline patients [32]. Web-based word-of-mouth information about physicians obtained by patients before their visit helps patients to know the physicians better, such as the physicians’ manner, treatments, and knowledge. Therefore, web-based word-of-mouth information can reduce patients’ perceived scarcity of information about their physician’s medical services, leading to our second hypothesis: patients’ perceived web-based word-of-mouth information about physicians reduces patients’ perceived scarcity of information regarding the physicians’ medical services (H2).

### Fear of Physicians’ Opportunistic Behaviors

In the principal-agent relationship, both parties expect to maximize their own interests [16]. The agents will work to increase their benefits, but some of their behaviors may even increase principals’ costs, leading to agents’ opportunistic behaviors [33]. As principals, patients are concerned about whether the physicians have opportunistic behaviors because patients cannot accurately evaluate physicians’ behaviors, especially in China. Owing to the imperfections of the medical systems in China, opportunistic behaviors of medical service providers have caused widespread concerns [34,35], such as whether physicians receive kickbacks, prescribe high-priced drugs [36], or ask patients to do excessive or unnecessary examinations or treatments [36], all of which are beneficial to physicians’ own interests but harm patients’ interests [37]. Opportunistic behaviors are also harmful to the physician-patient relationship because these behaviors reduce patients’ trust in physicians [38].

Patients can not only obtain health information such as diagnoses and treatments through eHealth data but also browse web-based reviews about physicians. Compared with offline word-of-mouth information, web-based word-of-mouth information has a greater impact on consumers’ behaviors because of its extensive sources, large coverage, and convenient dissemination [39]. Positive web-based word-of-mouth information can effectively reduce principals’ concerns about agents’ opportunistic behaviors [24,40]. Web-based word-of-mouth information about physicians helps improve the transparency of medical services and enhance patients’ confidence in medical decisions [41]. It also reflects the experiences of other patients with similar diseases [42]. With more web-based word-of-mouth information about the physicians, patients can evaluate the likelihood of the physicians’ opportunistic behaviors, and then they can choose physicians who are less likely to engage in those opportunistic behaviors. Therefore, positive web-based word-of-mouth information helps reduce patients’ concerns about physicians’ opportunism. Physicians’ opportunism, in this study, is defined as the behaviors of physicians who do not provide good services but charge high prices, conduct excessive and unnecessary examinations, and receive rebates to prescribe high-priced drugs [36]. With better web-based word-of-mouth information about physicians, patients will be less apprehensive of the physicians’ opportunistic behaviors, leading to the following hypothesis: patients’ perceived web-based word-of-mouth information about a physician reduces patients’ fear of the physician’s opportunism (H3).

### Trust

In the principal-agent relationship, trust is the most valuable aspect [43], because if the relationship occurs under ideal conditions, there is no need for trust [44,45]. Trust is the expectation that an individual or a group will make an effort of good faith to behave following commitments (both explicit and implicit), to be honest, and not to take excessive advantage of others, even when the opportunity exists [46]. Owing to the scarcity of patients’ information about clinical diagnoses and treatments, the asymmetry of physicians’ medical service information between patients and physicians makes it difficult for patients to determine whether the physicians are trustworthy [24]; therefore, in the physician-patient relationship, the information scarcity of physicians’ medical services impedes patients’ trust in the physicians, leading to hypothesis 4: patients’ perceived information scarcity about physicians’ medical service information reduces patients’ trust in physicians (H4).

In the principal-agent relationship, as agents, patients’ fear of physicians’ opportunistic behaviors also influences patients’ trust in physicians. Existing research has confirmed that opportunistic behavior in web-based banking leads to low levels of trust of users in internet banking [47]. In the e-commerce environment, fear of sellers’ opportunism also harms buyers’ trust [33]. In the physician-patient relationship, opportunistic behaviors are also harmful because these behaviors reduce patients’ trust in physicians [38]. Although physicians’ behaviors are not always immoral, patients still worry about the possibility of physicians’ opportunistic behaviors because the principal-agent relationship is favorable for physicians to act immoral behaviors. This worry will be enhanced if the possibility of the physicians’ opportunism is high. Physicians’ opportunistic behavior benefits their own interests but harms the interests of patients, which also impedes patients’ trust in them. Patients cannot monitor physicians’ behaviors, and they worry that their physicians will act opportunistic behaviors; thus, the fear of physicians’ opportunism reduces patients’ trust, leading to hypothesis 5: the fear of physicians’ opportunism reduces patients’ trust in physicians (H5).

Web-based word-of-mouth information is an important factor affecting potential customers’ purchase intentions and behaviors [48,49], because web-based word-of-mouth information reflects previous consumers’ evaluation of the products. In medical situations, some studies have also explored the impact of web-based physician reviews on patients’ decision-making behavior. For example, higher web-based ratings of physicians increase patients’ intention to consult them [50]. Web-based word-of-mouth information about physicians also increases physicians’ offline visits [51]. Acting as the previous patients’ evaluation cue, physicians’ web-based word-of-mouth information serves as an important reference for the selection of physicians by patients. The better the patients perceive web-based word-of-mouth information about the physicians, the more favorable it is for the patients to trust in the physicians, leading to the following hypothesis: patients’ perceived web-based word-of-mouth information about physicians increases patients’ trust in the physicians (H6).

### URT Overview

In the principal-agent relationship, uncertainty arises because the principal cannot fully monitor the agent’s behavior, resulting in adverse selection [17] and the moral hazard of hidden behaviors [18]. It is important to understand how to reduce uncertainty in this relationship. For example, reducing uncertainty can increase consumers’ purchase intention and lead to an actual purchase [24]; reducing uncertainty can also increase users’ trust in the web-based world so that they can effectively use a tool [52]. Originating from the field of interpersonal communication, the URT posits that uncertainty occurs when people cannot predict the future behavior of others or when they do not meet their own expectations [52,53]. URT is widely used in fields such as organizational behavior and information systems, among others [52]. For example, Srivastava and Chandra [52] considered 3 ways to reduce users’ uncertainty to enhance their trust and use intention in the web-based world. The three ways include acquiring information passively through observation, acquiring information actively through third-party search, digital signatures, and third-party authentication, and acquiring information from interactions, such as direct interaction with the target object [52].

In the medical scenario, patients’ uncertainties, that is, their inability to accurately predict the state of their disease because of a lack of information, exist in every aspect of their diagnoses and treatments. Uncertainties in the principal-agent relationship are caused by specific information problems (eg, hiding information and hiding behavior), and these problems can be alleviated by the use of information systems [24]. In the physician-patient relationship discussed previously, the disclosure of information comes from the agent (eg, medical information provided by the physician), and it reduces only a few uncertainties of patients, but with the development of technology, medical and health information is no longer only in the hands of the medical providers (agents). The client can actively acquire medical and health information from a third party [20], enabling patients to overcome the restrictions of time and space and actively obtain information about the causes of diseases, treatments, and reputations of physicians and hospitals through the internet. With the active information acquisition method [52] to reduce uncertainty, patients’ web-based search behavior can help actively reduce uncertainty, but the influence mechanism of how web-based information acquired by patients reduces uncertainty is not yet clear.

### Patients’ Uncertainty

Uncertainty in the medical context refers to a cognitive state in which the meaning of medical events cannot be determined [3,4]. Uncertainty, as a medical experience characterized by unpredictability, unfamiliarity, and ambiguity, is associated with poor medical outcomes and psychological states (eg, fear, stress, and loss of control) [43]. Existing research on uncertainties in the medical field has mainly focused on information uncertainty related to diseases, diagnoses, and treatments [5]. Uncertainties regarding illness can be divided into the medical providers’ uncertainty about diseases and the patients’ uncertainty about diseases. Previous research has mainly focused on the physician’s uncertainty of expressing disease-related information during patients’ visits and its impact [54]. The latter, that is, patients’ uncertainty about diseases, is the focus of this study.

Patients’ uncertainty means that the patients are unable to determine the meaning of disease-related events or accurately predict the outcomes of such events [5,6]. In the uncertainty in illness theory presented by Mishel [3], the antecedents (eg, symptom stimulus, patients’ cognitive abilities, and physicians’ information authorities), the appraisal process, the coping mechanism, and the adaptation outcomes of patients’ uncertainty in diseases are concluded, and the scale of patient uncertainty about illness is developed. This theory is effective in guiding interventions to manage patients’ uncertainty [55].

In web-based markets, as sellers cannot fully describe the product or predict the products’ future performances, consumers’ uncertainty about products and sellers should be distinguished, between which the former uncertainty is related to the description and performance of products, and the latter uncertainty is related to sellers’ adverse selection and moral hazard [15]. The uncertainty about sellers also increases uncertainty about products, and the two types of uncertainties reduce price premiums [15]. Similarly, in the physician-patient relationship, as physicians cannot fully describe the diseases or predict the effectiveness of treatments, patients’ uncertainty in the process may be not only about the diseases but also about the physicians. Owing to the traits of principal-agent relationship, patients, who are the inferior party because of the scarcity of information, tend to question the rationality of physicians’ advised medical treatments. However, in the medical context, few researchers have focused on patients’ uncertainty about physicians. Given this, considering the principal-agent relationship between physicians and patients, we follow the classification of customers’ uncertainties about sellers and products by Dimoka et al [15] to distinguish between patients’ uncertainty about diseases and patients’ uncertainty about physicians. In this way, this study can contribute to research on patients’ uncertainty.

In the principal-agent relationship, how much information principals have played a key role in their uncertainty [23,24]. The lower the availability of product information, the greater the consumers’ uncertainty about the product quality [23]; therefore, in our context, patients’ perception of scarcity of disease information can increase patients’ uncertainties about the diseases, and we hypothesize the following: patients’ perceived information scarcity about diseases increases patients’ uncertainty about the diseases (H7).

Owing to information scarcity, it is difficult for patients to judge the quality of physicians’ medical services. Less information about physicians’ medical services leads to patients’ stronger sense of uncertainty about physicians. According to research on the uncertainty of patients regarding disease [3], the causative factors include event familiarity. When patients have more knowledge about the physicians’ medical services, it helps to reduce their uncertainty about the physicians’ medical services, leading to the following hypothesis: patients’ perceived information scarcity about physicians’ medical services information increases patients’ uncertainty about the physicians (H8).

Trust can overcome uncertainty, and trust is necessary only when the environment is uncertain [45]. When patients trust their physicians, they can predict their physicians’ behaviors based on their belief in the physicians’ integrity, benevolence, and competence under uncertain circumstances. They believe that their physicians are honest and have great capabilities. Therefore, this study believes that a patient’s trust in a physician will help reduce the patient’s uncertainty about the physician, leading to hypothesis 9: a patient’s trust in a physician can mitigate the patient’s uncertainty in that physician (H9).

Because of the internally inconsistent goals between physicians and patients, physicians’ opportunistic behaviors are inevitable, such as physicians taking kickbacks to prescribe expensive drugs, unnecessary tests, and overtreatment. Patients often lack professional information to judge the rationality of physicians’ treatment plans and examination procedures, which leads to a sense of uncertainty about the rationality of physicians’ treatment behaviors. In China, concern about physicians’ opportunistic behavior is an important factor that leads to patients’ sense of uncertainty [37,56]. Possible opportunistic behavior of vendors’ drug prescription also leads to more uncertainty for buyers [45], and thus we hypothesize the following: patients’ fear of the physician’s opportunism increases patients’ uncertainty in the physician (H10).

Consumers’ uncertainty about sellers is distinct from the uncertainty about products, between which the former uncertainty can increase the latter uncertainty [15]. The process of patient consultation entails providing a series of examinations, diagnoses, and other services by the physician to identify the disease and determine other relevant treatments for the patient. If patients are uncertain about the rationality of the medical services provided by physicians and doubt the rationality of the physician’s examination and treatment plans, it will be detrimental to patients’ certainty about the disease; therefore, we hypothesize the following: patients’ uncertainty about the physician increases patients’ uncertainty about their diseases (H11).

**Figure 1:**
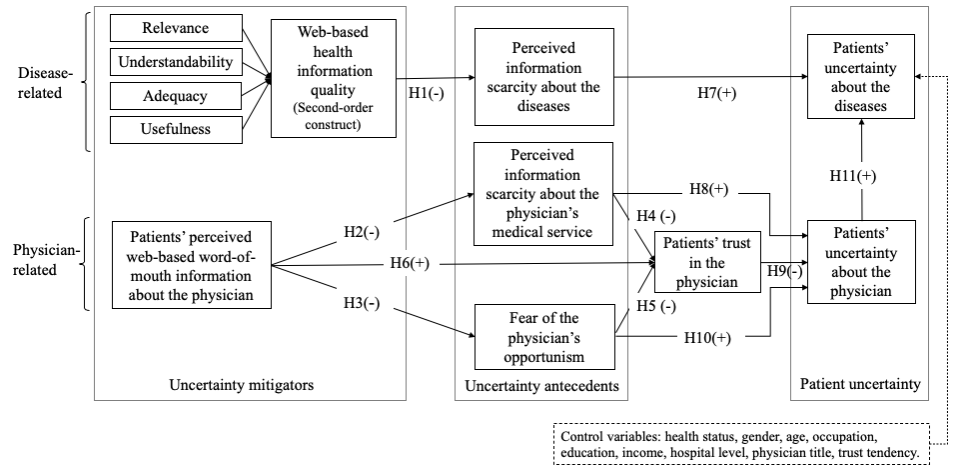
Research conceptual model. H: hypothesis.

In summary, based on existing literature, this study uses the principal-agent theory and the URT to develop a research model to explain the mechanism of how web-based information search by patients can reduce their uncertainties, as shown in Figure 1. In the context of patients’ active information acquisition, we hypothesize that two types of web-based health information (ie, web-based disease-related information quality and web-based physician-related information) as uncertainty mitigators to reduce patients’ uncertainty. When identifying the antecedents of uncertainty and its consequences, we followed the descriptions by Pavlou et al [24] and Srivastava and Chandra [52] on the use of unique and specific variables related to customers’ uncertainty. Principals’ perception of information scarcity and principals’ concern about agents’ opportunistic behaviors are the causes of the principals’ uncertainty. Principals’ trust in the agents acts as a mediator when the uncertainty antecedents reduce principals’ uncertainty. Following the classification of customers’ uncertainty about sellers and products by Dimoka et al [15], we classified patients’ uncertainties into uncertainty about diseases and uncertainty about physicians. Our research model is depicted in Figure 1.

## Methods

### Ethical Considerations

An ethics review was not applicable for this study because the online survey measured the subjects’ perceptions and did not influence their perceptions or attitudes.

### Data Collection

This study adopted the survey method to collect data. A total of 108 questionnaires were collected for the pilot test before the formal survey. The wording of some items and typesetting in the questionnaire were modified according to the feedback of the participants. A professional survey company (Wenjuanxing) was responsible for collecting the formal data. The survey started in May 2020 and lasted for a month. Each questionnaire corresponding to a separate IP address provided a reward of RMB 14 (US $2.20). The questionnaire was also set to ensure that valid respondents should answer all the questions before submitting. At the beginning of the questionnaire, the background of the survey was introduced, and screening questions were set to meet the 3 requirements for the survey. Only those who might have a certain disease and have seen a physician offline within 3 months, who had engaged in web-based disease information search behavior, and who had read the web-based word-of-mouth information of the visited physicians were eligible.

Specifically, our questionnaire first used 3 questions to exclude invalid respondents. The first question was “Did you suffer a certain disease and have any experience of offline medical treatments in the past three months?” The respondents who answered “Yes” proceeded to the next question, and the respondents who answered “No” were regarded as invalid respondents, and their questionnaires were terminated. Then, at the top of each page, there was a statement “Please recall the most recent experience of seeing a physician within the past three months, and based on this experience, answer the following questions.” The second question to screen out the invalid respondents was “Before the consultation, have you searched for disease-related information on the internet for this consultation?” Similarly, respondents who answered “No” were prompted to end answering the questionnaire, and those who answered “Yes” continued to the next question. The third screening item was “Do you know the evaluation of the physician (based on the most recent visit within three months) on the Internet?” Only respondents who answered “Yes” continued to answer the questions about the perception of web-based word-of-mouth information, and respondents who answered “No” were prompted to end answering the questionnaire.

To ensure that the respondents responded seriously, the question regarding the evaluation of physicians’ medical services appeared twice in different places in the questionnaire. Questionnaires with completely inconsistent answers (eg, strongly disagree and very agree) were excluded. A total of 40 invalid respondents were screened out, and the final sample size was 337. This sample size meets the requirement that the sample size should be 5-10 observations for each estimated parameter [57,58].

### Measurements

All items in this study are from mature scales, as shown in Table 1. Web-based information quality is a formative construct and the measurements were from Zahedi and Song [59]. The modified scale for perceived web-based word-of-mouth information about physicians was derived from Collins and Stevens [60]. As mentioned previously, there is a filter item—“Do you know the evaluation of the physician (based on the most recent visit within three months) on the Internet?”—which asked the respondent whether he or she had browsed through the web-based word-of-mouth information about the physician from those who had previously consulted that physician. With this filter item, we could ensure that the respondent’s answers to web-based word-of-mouth information and other items were for the same physician. The measurement of perceived information scarcity about disease and physicians’ medical service was derived from Wells et al [23], who developed reflective measures to assess individuals’ degree of prepurchase information scarcity about products. The measurement of fear of physicians’ opportunism was from the measurement of fear of sellers’ opportunism [24,40], which referred to patients’ concerns about the rationality of the visited physicians’ treatment behaviors (eg, excessive examination and high-priced drugs). Patients’ trust measurement was modified from that suggested by McKnight et al [61] and Zhou et al [62]. Patients’ uncertainty about diseases was measured using the community scale of uncertainty in illness (Mishel Uncertainty in Illness Scale–Community form) [3]. Patients’ uncertainty about physicians was modified from the perceived uncertainty scale [63], which referred to patients’ uncertainty about the rationality of medical services provided by physicians. Respondents in this study are native Chinese speakers; therefore, all items were translated into Chinese. We conducted translation–back-translation procedure to ensure the validity of our questionnaire. Specifically, the translated questionnaire was evaluated by 2 doctoral students with relevant research backgrounds. Some adjustments were made to the wording and expression of the questionnaire based on their feedback. Items of constructs (ie, perceived web-based word-of-mouth information about physicians, perceived information scarcity, fear of the physician’s opportunism, perceived uncertainty, and trust) were measured by a 5-point Likert scale ranging from complete disagreement (1) to complete agreement (5). Items of the 4 dimensions of information quality were measured by the extent to which the internet health information conforms to the description in the item (eg, 1 point for a very low level and 5 points for a very high level).

**Table 1:** Construct measurement.

Construct, label, and source		Item
**IQ^a^ [59]**		
	Relevance1	For your health information needs, to what degree do you believe the internet health information provided by the website was applicable to your needs?
	Relevance2	For your health information needs, to what degree do you believe internet health information provided by the website was related to your needs?
	Relevance3	For your health information needs, to what degree do you believe internet health information provided by the website was pertinent to your needs?
	Relevance4	For your health information needs, to what degree do you believe internet health information provided by the website was relevant to your needs?
	Understandability1	For your health information needs, to what degree do you believe internet health information provided by the website was clear in meaning?
	Understandability2	For your health information needs, to what degree do you believe internet health information provided by the website was easy to read?
	Understandability3	For your health information needs, to what degree do you believe internet health information provided by the website was easy to comprehend?
	Understandability4	For your health information needs, to what degree do you believe internet health information provided by the website was understandable?
	Adequacy1	For your health information needs, to what degree do you believe internet health Information provided by the website was sufficient?
	Adequacy2	For your health information needs, to what degree do you believe internet health information provided by the website was complete?
	Adequacy3	For your health information needs, to what degree do you believe internet health information provided by the website was adequate?
	Adequacy4	For your health information needs, to what degree do you believe internet health information provided by the website contained the necessary topics or categories?
	Usefulness1	For your health information needs, to what degree do you believe internet health information provided by the website was informative?
	Usefulness2	For your health information needs, to what degree do you believe internet health information provided by the website was valuable?
	Usefulness3	For your health information needs, to what degree do you believe internet health information provided by the website was helpful?
	Usefulness4	For your health information needs, to what degree do you believe internet health information provided by the website was useful?
**PWOM^b^ [60]**		
	PWOM1	In online reviews, the physician is very popular and many patients come to see the physician.
	PWOM2	In online reviews, patients who visited the physician had a good experience.
	PWOM3	According to online reviews, the physician is a good physician.
	PWOM4	According to online reviews, the physician has a good relationship with patients.
**PSD^c^ [23]**		
	PSD1	I have a good idea of the disease-related information (eg, symptoms, causes of disease, treatment methods, etc).
	PSD2	I have sufficient information about the disease (eg, symptoms, cause of disease, treatment, etc).
	PSD3	I possess adequate knowledge about the disease-related information (eg, symptoms, causes of disease, treatment methods, etc).
**PSPMS^d^ [23]**		
	PSPMS1	I have a good idea of the medical services of the physician whom I visited this time.
	PSPMS2	I have sufficient information about the medical services of the physician for this visit.
	PSPMS3	I possess adequate knowledge about the medical service information of the physician whom I visited this time.
**FPO^e^ [24]**		
	FPO1	In this visit, the physician might not have provided good service but charged a high price.
	FPO2	In this visit, the physician might have overexamined, unnecessarily examined, or overtreated me.
	FPO3	In this visit, the physician might have received a rebate for prescribing an overpriced drug (eg, imported drug).
	FPO4	In this visit, the physician might have breached formal or informal agreements to his or her benefit.
**T^f^ [61,62]**		
	T1	The physician is sincerely concerned about my medical issues
	T2	The physician is honest in his or her medical practices
	T3	I believe that the physician does a very good job
	T4	I feel that I can count on the physician to help me with my medical problems
**MUIS^g^ [3]**		
	MUIS1	I don’t know what is wrong with me
	MUIS2	I have a lot of questions without answers.
	MUIS3	It is difﬁcult to know if the treatments or medications I am getting are helping.
	MUIS4	Because of the unpredictability of my illness, I cannot plan for the future.
	MUIS5	The effectiveness of the treatment is undetermined.
**PU^h^ [63]**		
	PU1	I think the rationality of the medical services provided by the physician involves a high degree of uncertainty.
	PU2	I think the rationality of the medicine prescribed by the physician is uncertain.
	PU3	I think the rationality of the disease examination and treatment plan is uncertain.
	PU4	The rationality of the services provided by the physician is uncertain (ie, the service I received may not be exactly what I wanted).
	PU5	I feel the uncertainty associated with the rationality of the medical services provided by the physician is high.
**TD^i^ [64]**		
	TD1	I generally trust other people.
	TD2	I generally have faith in humanity.
	TD3	I feel that people are generally reliable.
	TD4	I generally trust other people unless they give me reasons not to.

^a^IQ: web-based health information quality.

^b^PWOM: perceived web-based word-of-mouth information about physicians.

^c^PSD: perceived information scarcity about the diseases.

^d^PSPMS: perceived information scarcity about the physicians’ medical services.

^e^FPO: fears of physician’s opportunism.

^f^T: patients’ trust in the physician.

^g^MUIS: patients’ uncertainty about diseases.

^h^PU: patients’ uncertainty about the physician.

^i^TD: trust tendency.

To reduce other possible influences on our model, we considered control variables in 3 ways, although these variables are not our interest in this study. To reduce the possible influence of individual differences, demographic information, such as gender, age, education level, income per month, and occupation, is controlled. To reduce the possible influence of the impact of medical treatment, health-related and medical experience-related factors are also controlled, such as the respondent’s health status, the physician’s title (an official certification of a physician’s quality by the government) [65], and the hospital’s level (an official certification of a hospital’s quality by the government) [65]. To reduce the possible influence of the respondent’s characteristic of trust, the respondents’ trust tendency was also controlled. For example, with the same word-of-mouth information about a physician, some patients may easily trust the physician, whereas others may still doubt the physician. Trust tendency [64] was also measured by a 5-point Likert scale ranging from complete disagreement (1) to complete agreement (5).

## Results

### Overview

As the model measured in this study has a formative construct, partial least squares (PLS) structural equation modeling is suitable for data analysis. SmartPLS (version 3.3.3, SmartPLS GmbH) software was used in this study. In addition, PLS is also widely used in information systems research owing to its relaxed requirements for the normal distribution of samples, its ability to process data with small sample size, and its applicability to development theory rather than test theory [66]. We first used SmartPLS (version 3.3.3, SmartPLS GmbH) software to test the reliability and validity of the measurement model and then tested the path coefficients of the structural model.

### Descriptive Statistics

The respondents’ demographic information, health-related information, and medical experience–related information are shown in Table 2. More respondents were female (231/337, 68.5%). In terms of age distribution, age groups 21-30 years (165/337, 49%) and 31-40 years (117/337, 34.7%) were the most represented. Education level was relatively high, with high school and below accounting for only 4.5% (15/337). The monthly income distribution was relatively even. The surveyed samples were mainly working people, with enterprise employees accounting for 68.2% (230/337). The physicians’ titles and hospitals’ levels are also relatively high.

Table 3 lists the descriptive statistics of the constructs involved in the model.

**Table 2:** Demographic profile, health-related information, and medical experience–related information (N=337).

Characteristic		Value, n (%)
**Gender**		
	Female	231 (68.5)
	Male	106 (31.5)
**Age (years)**		
	18-20	23 (6.8)
	21-30	165 (49)
	31-40	117 (34.7)
	41-50	32 (9.5)
**Education**		
	Postgraduate or above	25 (7.4)
	Undergraduate	246 (73)
	3-year college	51 (15.1)
	High school	11 (3.3)
	Middle school or below	4 (1.2)
**Monthly income (RMB [US $])**		
	≤3000 (471.60)	55 (16.3)
	3000-5999 (471.60-943.20)	40 (11.9)
	6000-8999 (943.20-1414.80)	87 (25.8)
	9000-11,999 (1414.80-1886.40)	85 (25.2)
	12,000-14,999 (1886.40-2358)	43 (12.8)
	≥15,000 (2358)	27 (8)
**Occupation**		
	Student	46 (13.6)
	Enterprise worker	230 (68.2)
	Civil servant	39 (11.6)
	Individual operator	15 (4.5)
	Others	7 (2.1)
**Health status**		
	Excellent	14 (4.1)
	Very good	56 (16.6)
	Good	124 (36.8)
	Fair	134 (39.8)
	Poor	9 (2.7)
**Physician’s title**		
	Assistant physician	103 (30.6)
	Associate physician	123 (36.5)
	Chief physician	87 (25.8)
	Not sure	24 (7.1)
**Hospital’s level**		
	Primary hospital	52 (15.4)
	Intermediate hospital	72 (21.4)
	Senior hospital	203 (60.2)
	Not sure	10 (3)

**Table 3:** Descriptive statistics^a^.

Construct and item		Minimum value	Maximum value	Mean (SD)
**PWOM^b^**				
	PWOM1	2	5	4.04 (0.66)
	PWOM2	1	5	4.16 (0.80)
	PWOM3	1	5	4.08 (0.88)
	PWOM4	1	5	4.09 (0.85)
**PSD^c^**				
	PSD1	1	5	2.38 (0.83)
	PSD2	1	5	2.62 (1.03)
	PSD3	1	5	2.48 (0.97)
**PSPMS^d^**				
	PSPMS1	1	5	2.24 (0.76)
	PSPMS2	1	5	2.25 (0.90)
	PSPMS3	1	5	2.23 (0.87)
**FPO^e^**				
	FPO1	1	5	2.44 (1.04)
	FPO2	1	5	2.40 (1.19)
	FPO3	1	5	2.06 (1.07)
	FPO4	1	5	1.90 (1.08)
**T^f^**				
	T1	1	5	4.03 (0.76)
	T2	2	5	4.12 (0.78)
	T3	1	5	4.02 (0.79)
	T4	1	5	4.10 (0.81)
**MUIS^g^**				
	MUIS1	1	5	2.27 (0.94)
	MUIS2	1	5	2.70 (1.12)
	MUIS3	1	5	2.60 (1.15)
	MUIS4	1	5	2.35 (1.14)
	MUIS5	1	5	2.64 (1.05)
**PU^h^**				
	PU1	1	5	2.63 (1.04)
	PU2	1	5	2.28 (1.13)
	PU3	1	5	2.32 (1.18)
	PU4	1	5	2.43 (1.13)
	PU5	1	5	2.34 (1.08)
**TD^i^**				
	TD1	1	5	3.73 (0.74)
	TD2	1	5	3.91 (0.78)
	TD3	1	5	3.72 (0.90)
	TD4	1	5	3.78 (0.91)

^a^The web-based health information quality is a formative construct; therefore, the details of this construct are described in the *Measurement Model* section.

^b^PWOM: perceived web-based word-of-mouth information about physicians.

^c^PSD: perceived information scarcity about the diseases.

^d^PSPMS: perceived information scarcity about the physicians’ medical services.

^e^FPO: fears of physician’s opportunism.

^f^T: patients’ trust in the physician.

^g^MUIS: patients’ uncertainty about diseases.

^h^PU: patients’ uncertainty about physicians.

^i^TD: trust tendency.

### Common Method Variance

As with all self-reported data, we should examine the potential common method variance. We follow the suggestions of Podsakoff et al [67] to minimize potential common method biases. First, we tried procedural remedies of Podsakoff et al [67]. To reduce respondents’ evaluation apprehension and avoid their answers being socially desirable, at the beginning of the questionnaire, we reminded them that their answers are anonymous and there are no right or wrong answers to our questions. All items in the questionnaire were designed in a random order to ensure that the measurement of predictor and criterion variables are psychologically separated for respondents. To ensure that the scale items are specific, concise, and clear, we also conducted the pilot test before the formal survey. We modify the wording according to the feedback of the participants to reduce ambiguity.

Second, the Harman single-factor test was conducted to diagnose whether the common method bias is a problem [68]. We ran an exploratory factor with all variables included [23]. The results showed that more than one factor can be extracted from the unrotated solution, and the variance contribution rate of the first factor was not more than 50% (23.7%), so there was no one single major factor that can reflect the majority covariance of all items, indicating that common method bias was not serious [57].

Moreover, based on our survey context and the suggestions of Podsakoff et al [67], we conducted a single-common-method-factor approach by controlling for the effects of a single unmeasured latent method factor to control the common method variance. Following Liang et al [69], we included in the PLS model a common method factor whose indicators included all the indicators of the constructs in this study. We calculated each indicator’s factor loadings and variances substantively explained by the construct and by the method factor. Multimedia Appendix 1 provides the detailed procedure and results [67,69,70]. As shown in Table S1 in Multimedia Appendix 1, most factor loadings of the method factor are insignificant. The average substantively explained variance of the indicators is 0.594, whereas the average method-based variance is 0.002. The ratio of substantive variance to method variance was 297:1, indicating the variance of each observed indicator explained by its substantive construct is substantially greater than the variance explained by the method factor. Therefore, based on the studies by Liang et al [69] and Williams et al [70], we further conclude that common method bias is not a serious problem in this study.

### Measurement Model

First, we tested the reliability and validity of the formative indicators (ie, web-based information quality). As web-based health information quality is a second-order formative construct, this study follows the method suggested by Wetzels et al [71]. In the structural equation model, four first-order reflective constructs (ie, information relevance, understandability, adequacy, and usefulness) point to the second-order constructive variable (information quality). A total of 16 items in the first order are taken as the measurement items of second-order constructs. PLS and Bootstrap were used to test the reliability and validity of the model and the outer weight of second-order formative constructs. First, the results of reliability and validity test of first-order reflective constructs showed information relevance (Cronbach α=.641; composite reliability [CR]=0.786; average variance extracted [AVE]=0.480), information understandability (Cronbach α=.726; CR=0.830; AVE=0.551), information usefulness (Cronbach α=.699; CR=0.816; AVE=0.526), and information adequacy (Cronbach α=.868; CR=0.910; AVE=0.717) all have good reliability and validity. Then, we tested the reliability and validity of the information quality of the second-order formative index, and the weight of the information quality (0.263, 0.314, 0.293, and 0.463) was >0.2 and significant at the level of *P*<.001, which passed the reliability and validity test of the formative construct. The variance inflation factors among all items were <2, satisfying the multicollinearity test, and the outer weight was significant and >0.2 [72].

Second, reflective indicators of this model were tested. We followed the methods suggested by Lewis et al [58] and Straub et al [73] to test the reliability and validity of the measurement model. The results are listed in Table 4. First of all, we tested the reliability of the constructs. The results show that the component reliability of each construct is >0.7 with good internal consistency [74,75]. The average variance extraction is also >0.5, which has good convergent validity [76]. In most cases, Cronbach α is >.7, and in all cases, the values are >0.6, which are within the acceptable range [66].

**Table 4:** Construct reliability and validity.

Construct and item			Item loading		Cronbach α		CR^a^		AVE^b^
**PWOM^c^**					.653		0.793		0.500
	PWOM1	0.703							
	PWOM2	0.695							
	PWOM3	0.712							
	PWOM4	0.689							
**PSD^d^**					.740		0.852		0.658
	PSD1	0.797							
	PSD2	0.832							
	PSD3	0.805							
**PSPMS^e^**					.695		0.830		0.620
	PSPMS1	0.758							
	PSPMS2	0.784							
	PSPMS3	0.820							
**FPO^f^**					.852		0.900		0.692
	FPO1	0.821							
	FPO2	0.811							
	FPO3	0.863							
	FPO4	0.831							
**PU^g^**					.890		0.919		0.694
	PU1	0.861							
	PU2	0.828							
	PU3	0.828							
	PU4	0.803							
	PU5	0.844							
**MUIS^h^**					.797		0.861		0.554
	MUIS1	0.663							
	MUIS2	0.772							
	MUIS3	0.790							
	MUIS4	0.706							
	MUIS5	0.783							
**T^i^**					.731		0.832		0.554
	T1	0.710							
	T2	0.759							
	T3	0.805							
	T4	0.699							
**TD^j^**					.760		0.846		0.580
	TD1	0.834							
	TD2	0.796							
	TD3	0.740							
	TD4	0.666							

^a^CR: composite reliability.

^b^AVE: average variance extracted.

^c^PWOM: perceived web-based word-of-mouth information about physicians.

^d^PSD: perceived information scarcity about the diseases.

^e^PSPMS: perceived information scarcity about the physicians’ medical services.

^f^FPO: fears of physician’s opportunism.

^g^PU: patients’ uncertainty about physicians.

^h^MUIS: patients’ uncertainty about diseases.

^i^T: patients’ trust.

^j^TD: trust tendency.

As shown in Table 5, we also tested the discriminant validity of the measurement model. The square root of the AVE (ie, italicized number on the diagonal line) for each factor in the table is larger than the correlation coefficient between the factor and other factors, so this measurement model has good discriminant validity [76]. Therefore, all the reflective constructs of this measurement model have good reliability and validity.

**Table 5:** Discriminant validity analysis^a^.

Construct	IQ^b^	PU^c^	PSPMS^d^	PWOM^e^	T^f^	FPO^g^	MUIS^h^	PSD^i^
IQ	—^j^	—	—	—	—	—	—	—
PU	−0.323	*0.833*	—	—	—	—	—	—
PSPMS	−0.506	0.258	*0.788*	—	—	—	—	—
PWOM	0.405	−0.336	−0.511	*0.700*	—	—	—	—
T	0.379	−0.539	−0.473	0.532	*0.744*	—	—	—
FPO	−0.154	0.711	0.118	−0.268	−0.380	*0.832*	—	—
MUIS	−0.365	0.678	0.301	−0.279	−0.497	0.507	*0.744*	—
PSD	−0.588	0.255	0.487	−0.273	−0.334	0.068	0.296	*0.811*

^a^The italicized values represent the square root of the average variance extracted for each construct.

^b^IQ: web-based health information quality.

^c^PU: patients’ uncertainty about the physician.

^d^PSPMS: perceived information scarcity about the physicians’ medical services.

^e^PWOM: perceived web-based word-of-mouth information about physicians.

^f^T: patients’ trust in the physician.

^g^FPO: fears of physician’s opportunism.

^h^MUIS: patients’ uncertainty about diseases.

^i^PSD: perceived information scarcity about the diseases.

^j^Not applicable.

### Construct Model and Results

**Figure 2:**
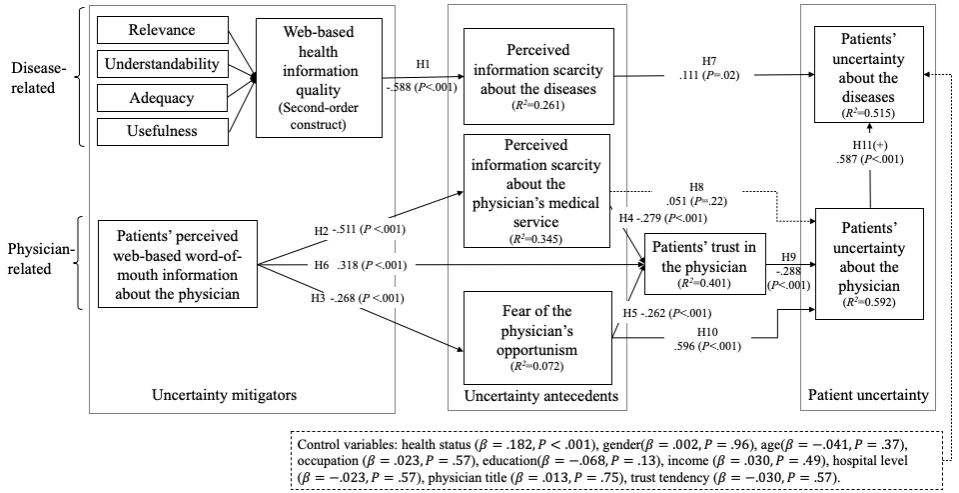
Structural equation model results. H: hypothesis.

We used PLS to test the hypotheses of this model and the Bootstrap method to test the significance of path coefficients [77]. The results are shown in Figure 2, and the path coefficients and T values are shown in Table 6. The control variables were also included in the model as predictors of the finally dependent variable (ie, patients’ uncertainty about the diseases). From Figure 2, the *R*^2^ of this model for patients’ uncertainty in diseases is 0.515. Both the disease- and physician-related uncertainty mitigators have significant effects on the uncertainty antecedents. Specifically, web-based health information quality can reduce patients’ perceived information scarcity about diseases (β=−.588; *P*<.001), supporting H1. Patients’ perceived web-based word-of-mouth information about physicians can reduce patients’ perceived information scarcity about the physician’s medical service (β=−.511; *P*<.001) and fears of physicians’ opportunism (β=−.268; *P*<.001), thus supporting H2 and H3. Patients’ perceived web-based word-of-mouth of physicians also increases patients’ trust in the visited physician (β=.318; *P*<.001), supporting H6.

**Table 6:** Hypotheses test results.

Hypothesis	Path	Path coefficient (SD)	*P* value	Result
H1	IQ^a^→PSD	−.588 (0.035)	<.001	Supported
H2	PWOM^b^→PSPMS^c^	−.511 (0.045)	<.001	Supported
H3	PWOM→FPO^d^	−.268 (0.048)	<.001	Supported
H4	PSPMS→T^e^	−.279 (0.062)	<.001	Supported
H5	FPO→T	−.262 (0.050)	<.001	Supported
H6	PWOM→T	.318 (0.068)	<.001	Supported
H7	PSD^f^→MUIS^g^	.111 (0.045)	.02	Supported
H8	PSPMS→PU^h^	.051 (0.045)	.22	Rejected but fully mediated by patients’ trust in the physician
H9	T→PU	−.288 (0.043)	<.001	Supported
H10	FPO→PU	.596 (0.047)	<.001	Supported and partially mediated by patients’ trust in the physician
H11	PU→MUIS	.587 (0.043)	<.001	Supported

^a^IQ: web-based health information quality.

^b^PWOM: perceived web-based word-of-mouth information of the physician.

^c^PSPMS: perceived information scarcity about the physicians’ medical services.

^d^FPO: fears of physician’s opportunism.

^e^T: patients’ trust in the physician.

^f^PSD: perceived information scarcity about the diseases.

^g^MUIS: patients’ uncertainty about diseases.

^h^PU: patients’ uncertainty about the physician.

Patients’ perceived information scarcity about the physician’s medical service reduces their trust in the visited physician (β=−.279; *P*<.001), supporting H4. Fear of physicians’ opportunism reduces patients’ trust in the visited physician (β=−.262; *P*<.001), supporting H5. Patients’ perceived information scarcity about the diseases increases patients’ uncertainty in diseases (β=.111; *P*=.02), supporting H7. However, patients’ perceived information scarcity about physicians’ medical services has no significant influence on patients’ uncertainty in the visited physician (β=.051; *P*=.22), thus rejecting H8. Patients’ trust in the visited physician can reduce patients’ uncertainty in the visited physician (β=−.288; *P*<.001), supporting H9. Fear of physicians’ opportunism has the most significant positive effect to increase patients’ uncertainty about the physician (β=.596; *P*<.001), supporting H10. Finally, uncertainty about the visited physician can increase patients’ uncertainty in diseases (β=.587; *P*<.001), supporting H11.

Besides respondents’ perception about their health status, other control variables have no significant influence on the model. Health status has a significantly negative impact on the model, which means that compared with patients who feel their health status is poor, patients who feel they are healthy perceive a higher level of uncertainty about the diseases.

To further explore the possible explanation of the rejection of H8, we conducted the Sobel test [78,79] to investigate the mediation role of trust in the relationship between the uncertainty antecedents and patients’ uncertainty about the physician. From the results in Table 7, after introducing patients’ trust in their physicians, the relationship between patients’ perceived information scarcity about physicians and their uncertainty about the physicians becomes nonsignificant, indicating that patients’ trust in their physicians fully mediates the relationship of H8; therefore, the direct relationship of H8 is rejected, and only when more information can increase patients’ trust, their uncertainty about physicians can be reduced. Moreover, increasing physicians’ medical service information can be effective in reducing patients’ uncertainty about their physicians. The relationship between patients’ fear of the physician’s opportunism and their uncertainty about the physician is still significant, indicating patients’ trust in their physicians partially mediates the relationship of H10. The Sobel test statistics [80] are also significant, which further confirms that patients’ trust in their physicians plays the role of mediation.

**Table 7:** The Sobel test of the mediating effect of patients’ trust in the physician.

Hypothesis and path				Path coefficient (SD)		Sobel test statistic		*P* value
**Hypothesis 8^a^**				N/A^b^		6.5734		<.001
	**PSPMS^c^→PU^d^**							
		Without mediator	.120 (0.054)		N/A		.02	
		With mediator	−.055 (0.052)		N/A		.29	
	PSPMS→T^e^		−.476 (0.053)		N/A		<.001	
	T→PU		−.492 (0.051)		N/A		<.001	
**Hypothesis 10^f^**				N/A		5.2280		<.001
	**FPO^g^→PU**							
		Without mediator	.656 (0.040)		N/A		<.001	
		With mediator	.579 (0.047)		N/A		<.001	
	FPO→T		−.380 (0.047)		N/A		<.001	
	T→PU		−.281 (0.041)		N/A		<.001	

^a^Fully mediated.

^b^N/A: not applicable.

^c^PSPMS: perceived information scarcity about the physicians’ medical services.

^d^PU: patients’ uncertainty about the physician.

^e^T: patients’ trust in the physician.

^f^Partially mediated.

^g^FPO: fears of physician’s opportunism.

## Discussion

### Principal Findings

This study investigated the mechanism of how web-based health information search behavior reduces patients’ uncertainty. Our empirical test results supported most of our hypotheses, except H8. Patients’ perceived web-based word-of-mouth information about physicians and the quality of web-based health information can effectively reduce patients’ uncertainty about diseases and physicians. The uncertainty reduction effect is achieved by affecting the antecedent factors of patients’ uncertainty, including patients’ fears of physicians’ opportunism, patients’ perceived information scarcity, and patients’ trust, which are all the traits of principal-agent relationship.

Specifically, the higher the possibility of the physician’s opportunism and information scarcity perceived by patients, the greater their uncertainty. Among the antecedents of patient uncertainty, patients’ fear of physicians’ opportunism has the most significant impact on patients’ uncertainty about physicians. By segmenting patients’ uncertainty, this research discussed the relationship between patients’ uncertainty about the diseases and patients’ uncertainty about physicians. The results show that patients’ uncertainty about physicians has a significant positive impact on patients’ uncertainty about diseases.

In addition, this study also demonstrated the significant role of patients’ trust in physicians. Patients’ perceived web-based word-of-mouth information about physicians can enhance patients’ trust in physicians. Patients’ having more information and less fear of physicians’ opportunistic behaviors also increases patients’ trust. However, from the result of the mediation test, only when the information can increase patients’ trust in their physicians, patients’ uncertainty about physicians can be reduced; thus, increasing physicians’ medical service information can be effective in reducing patients’ uncertainty about their physicians. Patients’ trust in their physicians fully mediates the relationship between their perception of information scarcity about the physicians’ medical service and their uncertainty about their physicians.

### Theoretical Contributions

First, on the basis the principal-agent theory and from the perspective of reducing patient uncertainty, this study is the first to explore the influence mechanism of web-based disease-related information quality and web-based word-of-mouth information received by patients on patients’ uncertainty. It is to be noted that information can reduce uncertainty, but the mechanism of how information reduces uncertainty is not clear. Therefore, we propose our *uncertainty mitigators–uncertainty antecedents–uncertainty* framework to explore the mechanism. On the basis of the URT, web-based information quality and web-based word-of-mouth information of physicians effectively reduce the antecedents of patients’ uncertainty, including perceived information scarcity, fears of physicians’ opportunism, and trust. Thus, patients’ uncertainty about the disease and the physician are reduced.

Second, this study enriches the literature on patients’ uncertainty by classifying patients’ uncertainties into patients’ uncertainty about the diseases and patients’ uncertainty about their physicians. Following the classification of consumers’ uncertainty about sellers and products by Dimoka et al [15], we also found that patients’ uncertainty about diseases and physicians should be distinguished. In particular, we explored the role of patients’ uncertainty about physicians, which has been rarely studied in the existing literature. Reducing patients’ uncertainty about their physicians can further reduce their uncertainty about diseases.

Third, this study emphasizes the significant role of patients’ trust. As an important factor in principal-agent relationships, trust is the most valuable aspect [43]. We also found that without trust, just increasing patients’ information does not help reduce their uncertainty about their physicians. This result further supports the fact that building trust is crucial to address the principal–agent problem.

### Practical Contributions

First, this study found that the better the web-based word-of-mouth information of a physician and information quality obtained by patients, the better the reduction in patients’ uncertainty. Therefore, for physicians in the internet era, attention should be paid to the role of web-based health information. More authoritative, more reliable, and higher-quality web-based platforms should be provided to meet patients’ demands for health information. In addition, physicians should encourage offline patients to participate in web-based word-of-mouth evaluations, maintain their own web-based word-of-mouth information, and provide more information about their services to potential patients [81]. Web-based word-of-mouth information can reach a wider audience and has a greater impact than offline word-of-mouth information. Web-based word-of-mouth information can effectively nudge physicians to improve their service quality and help patients acquire relevant information about physicians, thereby reducing patients’ uncertainty [39].

Second, from the full mediator role of trust, web-based information is effective only when this information can help build patients’ trust in their physicians. This suggests that web-based platforms that provide information (ie, web-based word-of-mouth information about physicians) should strictly check the quality of the information. More importantly, platforms can provide some cues to inform patients that the information is trustworthy, such as third-party certifications and guarantees. Only when patients can trust their physicians through this information can it help reduce their uncertainties.

### Limitations and Future Directions

First, as for the sample composition, there are 3 prerequisites for this study. Only those who might have a certain disease and had seen the physicians offline within the past 3 months, read the web-based word-of-mouth information about physicians, and engaged in web-based disease information search behaviors were eligible, which resulted in a large overrepresentation of younger people in our sample. More than 90.5% (305/337) of our respondents were aged <40 years, so the sample had possible self-selection bias and a bias of young age. Second, regarding the collection time of data, the research data were collected in May 2020 after the COVID-19 epidemic in China. The external validity of the results may be jeopardized. Then, this study only considered the influence mechanism of web-based word-of-mouth information about physicians on offline patients’ trust. Future studies can further consider the situation of web-based health consultation and investigate the possible differences in web-based health information on the physician-patient relationship in different channels. Moreover, because the focus of this study is the information about diseases and physicians, the respondents’ health status is controlled, and the result shows that respondents’ perception of their health status influences their uncertainty. Future studies can further discuss and explain the effect of health status. Finally, the study data were cross-sectional subjective data, which were provided by the same subjects at the same time, and future studies can use longitudinal analysis or experiments to better test the causal relationships in the model.

## Appendix

Multimedia Appendix 1 The procedure and results of common method variance.

## Abbreviations

AVE: average variance extracted
CR: composite reliability
PLS: partial least squares
URT: uncertainty reduction theory
