# Supplementary material for: The Mediating Role of Patients’ Trust Between Web-Based Health Information Seeking and Patients’ Uncertainty in China: Cross-sectional Web-Based Survey
Source: J Med Internet Res. 2022 Mar 11;24(3):e25275. doi: 10.2196/25275 (PMC8956986; doi:10.2196/25275)
Supplement: Multimedia Appendix 1 [file jmir_v24i3e25275_app1.docx]

**Appendix**

From Podsakoff et al. [67], when the predictor and criterion variables cannot be obtained from different sources and cannot measured in different contexts, and the source of the method bias cannot be identified and validly measured, the single-common-method-factor approach should be conducted to control the common method variance. The situation of this study is similar with the research of Liang et al. [69] and their research is the first that applied PLS to conduct the approach, so we refer to their approach using PLS to address the concern regarding common method bias.

Because the variance of each observed indicator is partitioned into trait, method, and random error, a latent method factor should be included in the structural model to assess method variance. We followed the procedure of Liang et al. [69] to convert each indicator to a single-indicator construct. Figure 1 is an example of converting indicators to single-indicator constructs of Liang et al. [69] and all constructs and the method factor become second-order constructs. In Figure 1, A is the independent variable and B is the dependent variable. a1, a2, b1, and b2 are indicators. e1 to e4 represent measurement errors. 𝜆1 to 𝜆4 are factor loadings. After the conversion, taking a1 as an example, when a1 is converted into a single-indicator construct A1, the measurement error of a1 becomes 0 and loading becomes 1. 𝜆1 becomes the path coefficient of A-A1. e1 becomes the error term of A1.

Following the approach, the PLS model for assessing common method bias of our study is depicted in Figure 2. The common method factor which links to all of the single-indicator constructs was included in the PLS model. For each single-indicator construct in Figure 2, we examined the coefficients and R square of its two incoming paths, including its substantive construct (e.g., a1) and the method factor. The results are listed in Table 1. By examining the statistical significance of factor loadings of the method factor and comparing the variances of each observed indicator explained by its substantive construct and the method factor can obtain the evidence of the common method bias [70]. From the results in Table 1, most of the loadings of the method factor are insignificant. The variance of each observed indicator explained by its substantive construct (R_S_^2^) is substantially greater than the variance explained by the method factor (R_M_^2^). These results can lead the conclusion that common method bias is not a serious concern in our study.


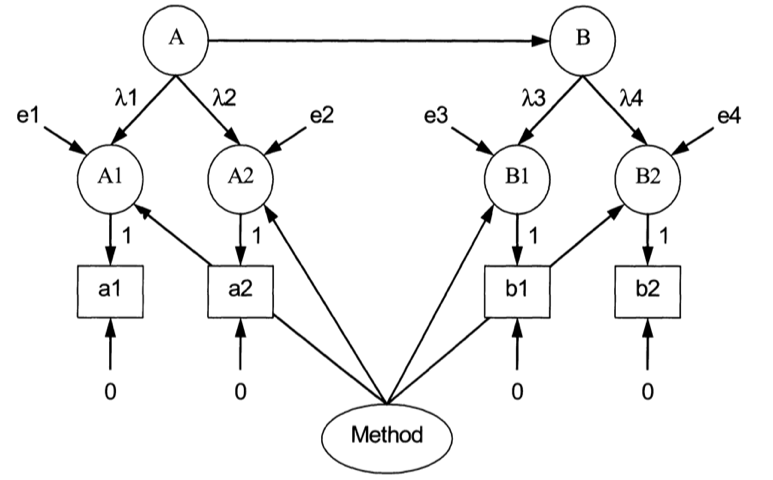


Figure 1. An example of converting indicators to single-indicator constructs [69].


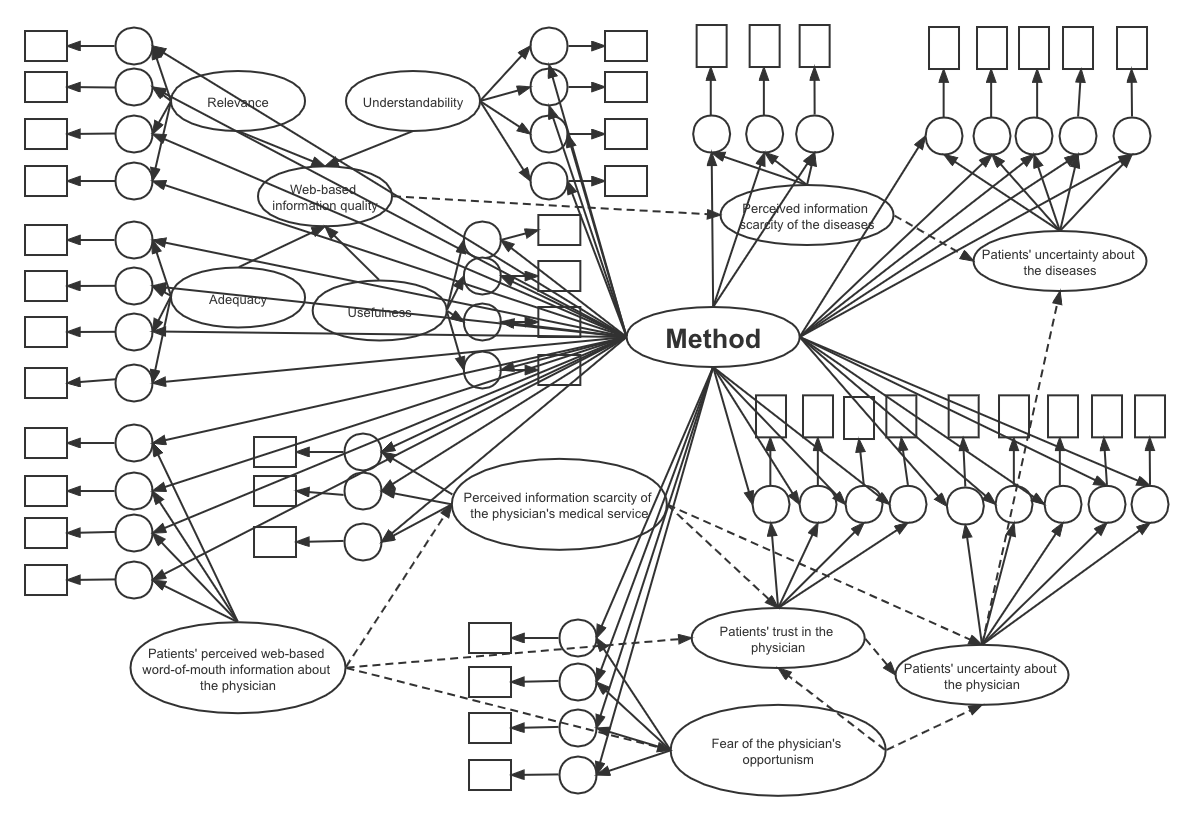


Figure 2. The PLS model to examine common method variance.

Table 1 Common Method Bias Analysis.

| Construct | Item | Substantive factor | | | Method factor | | | Total R^2^ |
| --- | --- | --- | --- | --- | --- | --- | --- | --- |
|  |  | Substantive factor loading | Standard deviation | R_S_^2^ | Method factor loading | Standard deviation | R_M_^2^ |  |
| IQ^a^ | Relevance1 | 0.761*** | 0.045 | 0.538 | 0.047 ^ns^ | 0.047 | 0.001 | 0.539 |
|  | Relevance2 | 0.720*** | 0.055 | 0.451 | 0.084 ^ns^ | 0.052 | 0.004 | 0.455 |
|  | Relevance3 | 0.664*** | 0.052 | 0.468 | -0.035 ^ns^ | 0.045 | 0.000 | 0.468 |
|  | Relevance4 | 0.633*** | 0.050 | 0.471 | -0.093 ^ns^ | 0.052 | 0.006 | 0.477 |
|  | Understandability1 | 0.836*** | 0.027 | 0.621 | 0.092* | 0.040 | 0.006 | 0.627 |
|  | Understandability2 | 0.818*** | 0.031 | 0.658 | 0.014^ns^ | 0.041 | 0.000 | 0.658 |
|  | Understandability3 | 0.617*** | 0.062 | 0.397 | -0.026^ns^ | 0.056 | 0.001 | 0.398 |
|  | Understandability4 | 0.682*** | 0.044 | 0.532 | -0.090* | 0.045 | 0.006 | 0.538 |
|  | Adequacy1 | 0.807*** | 0.033 | 0.683 | -0.036 ^ns^ | 0.040 | 0.001 | 0.684 |
|  | Adequacy2 | 0.815*** | 0.030 | 0.705 | -0.045 ^ns^ | 0.042 | 0.001 | 0.706 |
|  | Adequacy3 | 0.889*** | 0.026 | 0.744 | 0.048 ^ns^ | 0.038 | 0.002 | 0.746 |
|  | Adequacy4 | 0.874*** | 0.029 | 0.735 | 0.031 ^ns^ | 0.042 | 0.001 | 0.736 |
|  | Usefulness1 | 0.744*** | 0.041 | 0.538 | 0.025 ^ns^ | 0.041 | 0.000 | 0.538 |
|  | Usefulness2 | 0.759*** | 0.044 | 0.557 | 0.028 ^ns^ | 0.042 | 0.001 | 0.558 |
|  | Usefulness3 | 0.708*** | 0.034 | 0.540 | -0.061 ^ns^ | 0.040 | 0.003 | 0.543 |
|  | Usefulness4 | 0.688*** | 0.048 | 0.468 | 0.009 ^ns^ | 0.045 | 0.000 | 0.468 |
| PWOM^b^ | PWOM1 | 0.708*** | 0.049 | 0.499 | 0.003 ^ns^ | 0.056 | 0.000 | 0.499 |
|  | PWOM2 | 0.652*** | 0.053 | 0.458 | -0.042 ^ns^ | 0.052 | 0.001 | 0.459 |
|  | PWOM3 | 0.693*** | 0.047 | 0.512 | -0.038 ^ns^ | 0.052 | 0.001 | 0.513 |
|  | PWOM4 | 0.747*** | 0.048 | 0.492 | 0.078 ^ns^ | 0.048 | 0.004 | 0.496 |
| IAI^c^ | IAI1 | 0.792*** | 0.032 | 0.641 | 0.016^ns^ | 0.040 | 0.000 | 0.641 |
|  | IAI2 | 0.821*** | 0.026 | 0.689 | 0.016^ns^ | 0.036 | 0.000 | 0.689 |
|  | IAI3 | 0.821*** | 0.030 | 0.644 | -0.032^ns^ | 0.039 | 0.001 | 0.645 |
| HSAI^d^ | HSAI1 | 0.723*** | 0.041 | 0.564 | 0.048^ns^ | 0.044 | 0.001 | 0.565 |
|  | HSAI2 | 0.840*** | 0.031 | 0.650 | -0.056^ns^ | 0.039 | 0.002 | 0.652 |
|  | HSAI3 | 0.800*** | 0.029 | 0.650 | 0.010^ns^ | 0.043 | 0.000 | 0.650 |
| FPO^e^ | FPO1 | 0.920*** | 0.031 | 0.714 | -0.122** | 0.045 | 0.009 | 0.723 |
|  | FPO2 | 0.779*** | 0.033 | 0.654 | 0.047^ns^ | 0.045 | 0.001 | 0.655 |
|  | FPO3 | 0.903*** | 0.027 | 0.756 | -0.054^ns^ | 0.036 | 0.002 | 0.758 |
|  | FPO4 | 0.723*** | 0.036 | 0.647 | 0.132** | 0.048 | 0.010 | 0.657 |
| MUIS^f^ | MUIS1 | 0.681*** | 0.070 | 0.435 | -0.028^ns^ | 0.073 | 0.000 | 0.435 |
|  | MUIS2 | 0.656*** | 0.052 | 0.583 | 0.142** | 0.058 | 0.009 | 0.592 |
|  | MUIS3 | 0.788*** | 0.048 | 0.628 | 0.006^ns^ | 0.056 | 0.000 | 0.628 |
|  | MUIS4 | 0.786*** | 0.058 | 0.498 | -0.105^ns^ | 0.063 | 0.005 | 0.503 |
|  | MUIS5 | 0.812*** | 0.047 | 0.627 | -0.027 ^ns^ | 0.059 | 0.000 | 0.627 |
| DP^g^ | DP1 | 0.779*** | 0.045 | 0.736 | 0.098^ns^ | 0.052 | 0.004 | 0.740 |
|  | DP2 | 0.838*** | 0.044 | 0.689 | -0.010^ns^ | 0.049 | 0.000 | 0.689 |
|  | DP3 | 0.807*** | 0.060 | 0.682 | 0.024^ns^ | 0.066 | 0.001 | 0.683 |
|  | DP4 | 0.842*** | 0.052 | 0.644 | -0.050^ns^ | 0.060 | 0.000 | 0.644 |
|  | DP5 | 0.902*** | 0.044 | 0.719 | -0.067 ^ns^ | 0.053 | 0.001 | 0.720 |
| T^h^ | T1 | 0.813*** | 0.048 | 0.535 | 0.113* | 0.052 | 0.006 | 0.541 |
|  | T2 | 0.676*** | 0.052 | 0.547 | -0.088 ^ns^ | 0.054 | 0.004 | 0.551 |
|  | T3 | 0.832*** | 0.039 | 0.661 | 0.026 ^ns^ | 0.049 | 0.000 | 0.661 |
|  | T4 | 0.656*** | 0.058 | 0.478 | -0.049 ^ns^ | 0.057 | 0.001 | 0.479 |
| Average R^2^ | | - | - | 0.594 | - | - | 0.002 | 0.596 |

^a^IQ : online health information quality.

^b^PWOM : perceived online word-of-mouth physician ratings.

^c^IAI : information asymmetry about disease.

^d^HSAI : information asymmetry about physicians’ medical services.

^e^FPO : fears of physician opportunism.

^f^MUIS : patient uncertainty about illness.

^g^DP : patient uncertainty about physician.

^h^T : patient trust.

^ns^*P*>.05, **P*<.05, ***P*<.01, ****P*<.001
